# Building Ontologies in DAML + OIL

**DOI:** 10.1002/cfg.233

**Published:** 2003-02

**Authors:** Robert Stevens, Chris Wroe, Sean Bechhofer, Phillip Lord, Alan Rector, Carole Goble

**Affiliations:** Department of Computer Science University of Manchester Oxford Road Manchester M13 9PL UK

## Abstract

In this article we describe an approach to representing and building ontologies advocated by the Bioinformatics and Medical Informatics groups at the University of Manchester. The hand-crafting of ontologies offers an easy and rapid avenue to delivering ontologies. Experience has shown that such approaches are unsustainable. Description logic approaches have been shown to offer computational support for building sound, complete and logically consistent ontologies. A new knowledge representation language, DAML + OIL, offers a new standard that is able to support many styles of ontology, from hand-crafted to full logic-based descriptions with reasoning support. We describe this language, the OilEd editing tool, reasoning support and a strategy for the language’s use. We finish with a current example, in the Gene Ontology Next Generation (GONG) project, that uses DAML + OIL as the basis for moving the Gene Ontology from its current hand-crafted, form to one that uses logical descriptions of a concept’s properties to deliver a more complete version of the ontology.


**Comparative and Functional Genomics**
*Comp Funct Genom* 2003; **4**: 133–141.
Published online in Wiley InterScience (www.interscience.wiley.com). **DOI:** 10.1002/cfg.233

**Conference Review**

### Building ontologies in DAML + OIL

Robert Stevens*, Chris Wroe, Sean Bechhofer, Phillip Lord, Alan Rector and Carole Goble
*Department of Computer Science, University of Manchester, Oxford Road, Manchester M13 9PL, UK*

*Correspondence to:
Robert Stevens, Department of Computer Science, University of Manchester, Oxford Road, Manchester M13 9PL, UK.
E-mail:
robert.stevens@cs.man.ac.uk*

Received: 11 November 2002
Accepted: 18 November 2002

### Abstract

**In this article we describe an approach to representing and building ontologies advocated by the Bioinformatics and Medical Informatics groups at the University of Manchester. The hand-crafting of ontologies offers an easy and rapid avenue to delivering ontologies. Experience has shown that such approaches are unsustainable. Description logic approaches have been shown to offer computational support for building sound, complete and logically consistent ontologies. A new knowledge representation language, DAML + OIL, offers a new standard that is able to support many styles of ontology, from hand-crafted to full logic-based descriptions with reasoning support. We describe this language, the OilEd editing tool, reasoning support and a strategy for the language's use. We finish with a current example, in the Gene Ontology Next Generation (GONG) project, that uses DAML + OIL as the basis for moving the Gene Ontology from its current hand-crafted, form to one that uses logical descriptions of a concept's properties to deliver a more complete version of the ontology.** **Copyright © 2003 John Wiley & Sons, Ltd.**

**Keywords:** **description logic; ontology; DAML + OIL; ontology development environment; ontology development methodology; reasoning**

### Introduction

In this article, we wish to present a style of building and delivering ontologies that we use in the Bioinformatics and Medical Informatics Groups at the University of Manchester. These groups have been advocates for the use of ontologies in their respective fields, and for associated technologies for their building, and management.

This article presents a view of ontologies built using a particular form of knowledge representation language, viz. description logic (DL). The Medical Informatics and Bioinformatics groups at the Manchester have a long history of building and using ontologies in the biomedical fields, notably in:

- The Galen [1] project, which demonstrated the use of DLs and their associated compositional approach to descriptions to deliver large-scale medical terminologies.
- TAMBIS [2] used a DL ontology to provide the illusion of a common query interface to multiple, diverse and distributed bioinformatics resources.
- MyGrid [3], which currently uses an ontology of bioinformatics services to discover, compose and semantically describe services available on the web.
- The Gene Ontology Next Generation (GONG) [4], which uses a DL to migrate the Gene Ontology (GO)[5] to an explicitly defined form that delivers a more complete and robust ontology.

Running through all these projects is the theme of DLs as a knowledge representation language. In this article we argue why we think this particular representation is good for describing biomedical knowledge and why one representation in particular, DAML + OIL, is particularly well suited for this purpose.

Many of the resources used within biomedicine contain not only data in the form of biological

Copyright © 2003 John Wiley & Sons, Ltd.

sequences, clinic attendance dates, temperatures, etc. but also act as vast knowledge repositories. Much of this knowledge is stored in natural language as scientific writing. The volume and complexity of this knowledge means that human scientists increasingly need computational support to manage and analyse these data and exploit this knowledge. As long ago as 1893, the British medical establishment decided it needed a controlled vocabulary describing 'why people die', in order to ease the generation of statistical data. So was born the International Classification of Diseases (ICD). ICD 10 [6] how contains over 15 000 terms, held within a classification, representing diseases, symptoms, conditions, problems, complaints and other reasons for the provision of a medical service or procedure. Similarly, bioinformatics has seen the need for controlled vocabularies, such as the SWISS-PROT keywords (**http://www.expasy.ch/cgi-bin/keywlist.pl**) [8], to index its database entries and ease retrieval. Recent activities in bioinformatics, such as GO [5], have seen the use of ontologies as a means of delivering controlled vocabularies.

Traditionally, in these terminologies each concept or term has been placed by hand within the classification. Experience has shown that this approach, while an easy and attractive method to start with, is fraught with dangers [7]. In a classification where any particular term may have many parents and many children, it is all too easy to omit parents or to place a new term incorrectly. Such errors will leadnot only to an incorrect description of the domain, but to errors in recall and precision of queries and inaccurate statistics of phenomena.

If the hand-crafted approach has its drawbacks, can the computer science community offer a better solution? Any solution needs to be usable by biologists, who are by far the best people to build ontologies of biology.

The groups at Manchester would argue that DLs offer such a solution. This form of knowledge representation language does what the name suggests: the ontology is described with logic expressions. A reasoning engine can then process these logical expressions to produce a classification based on those descriptions and to find any contradictions in those descriptions. This means that, given suitable descriptions, a computer can build a taxonomy and place the concepts within that taxonomy at the correct place, according to the descriptions given.

Thus, there is a need for a computational approach to building ontologies. The approach we offer can deliver such support, but it should not exclude other, simpler approaches. Expert communities still need to be able to 'buy in' to the method we advocate.

The particular knowledge representation language we use, DAML + OIL, does not exclude the representation of simple, hand-crafted ontologies, so it is possible to migrate to full description-based ontologies, by increasing use of DAML + OIL's expressivity and reasoning support. As will be seen in this article, some problems still remain, but the wide spectrum of styles supported by DAML + OIL allows entry to this kind of technology at any level.

In this article we will describe the knowledge representation language we use to describe our ontologies, an example of tool support for writing DAML + OIL and the way reasoning is used to support the process of building our ontologies. We then present a general strategy to provide the infrastructure for building an ontology using a knowledge representation language such as DAML + OIL. Next we introduce a project, GONG, where we use DAML + OIL to add concept descriptions for terms from GO and thus begin the migration from a hand-crafted, phrase-based ontology to one couched in logical descriptions of those same concepts. Finally, we enter into a brief discussion of the work at Manchester and some of our future activities.

### Description logics and DAML + OIL

DAML + OIL is an ontology language specifically designed for the move from syntactic to full semantic interoperability of web-based resources [9]. We can already plumb resources together easily enough but, as different resources use different values to represent the same knowledge and the same values to represent different knowledge, this plumbing will not achieve the task of interoperation. Such interoperability relies on machine-interpretable semantic descriptions. DAML + OIL is underpinned by an expressive DL[10]. It is these formal semantics that enable machine interpretation and reasoning support and additionally aid human communication–an aim of ontological description.

Copyright © 2003 John Wiley & Sons, Ltd. 

DAML + OIL takes an object-oriented approach to modelling, with the structure of the domain being described in terms of concepts or in DAML + OIL's terms '*classes*' and '*properties*'. Properties are an explicit description of those attributes that enable class membership to be determined, e.g. a hydrolase has the property of catalysing hydrolysis, whereas one of a transcription factor's properties is to bind to DNA. An DL ontology consists of a set of *axioms* that assert, for example, subsumption ('kind-of') relationships between classes or properties. So, we can state an axiom that says that the concept 'enzyme' is a subclass of the concept 'protein'. We can also state an axiom that describes the concept 'enzyme' as having the property of 'catalyses reaction', i.e. a protein must catalyse a reaction in order to be an enzyme. Figure 1 shows a textual representation of a class or concept definition of some chemicals from the tricarboxylic acid cycle. A tricarboxylic acid is defined as a kind of organic acid that has three unionized carboxylic or carboxylate anion groups. Oxaloacetate is described as having three such groups, but malate is described as only having two such groups. By eye, we can reason that oxaloacetate is therefore a kind of tricarboxylic acid and malate is not. With DAML + OIL, a machine reasoner can use DAML + OIL's logical descriptions to work out the classification implied by those descriptions and any logical inconsistencies in those descriptions. Hence the name 'description logics' — logics that reason about the descriptions of the class.

All this expressivity may be used in class descriptions and can be used to capture medical, bioinformatics and molecular biology domain knowledge with high fidelity. However, it is also possible to simply assert class names within a taxonomic structure. Concept definitions can be as simple as possible yet as complex as necessary. Thus, DAML + OIL is capable of encoding a full range of ontologies, but its power lies in the possibility of formal description and the reasoning it can then support [11].

### Building ontologies with oiled

DAML + OIL provides us with a language for defining ontologies. Expecting users to model explicitly in DAML + OIL's underlying RDF(S)-based concrete syntax is not a viable option, so in order to fully exploit the expressiveness supported by the language and encourage the use of the language, tools are required that allow users to build and maintain these ontologies.

**Figure 1.** Some class descriptions for malate, oxaloacetate and tricarboxylic acid

Copyright © 2003 John Wiley & Sons, Ltd.

OilEd (**http://oiled.man.ac.uk**) [11,12] is a simple ontology editor that allows users to construct and manipulate DAML + OIL ontologies (Figure 2). It provides a graphical user interface separating the user from the underlying concrete syntax of DAML + OIL. In its current incarnation, OilEd is a relatively simple tool that does not support the full range of functionality that would be expected of an ontology engineering environment (such as versioning, change management, support for integration and merging). It does, however, support the full expressive power of DAML + OIL's concept language (note that OilEd's datatype support is minimal) [10], allowing the user to build ontologies using the full range of constructors, such as Boolean operators and explicitly quantified restriction types. The user is not *required* to model using complex constructs, though, and the tool can be used to construct simple taxonomies, which may then be further elaborated at a later date.

In spite of its limitations, OilEd has been used successfully in a number of academic projects [including GONG (**http://gong.man.ac.uk**); [10,13] see Section 5] and myGrid (**http://mygrid.man.ac.uk**) [14], as a tool for teaching in several university departments and for constructing ontologies within industry. OilEd is freely available for download under an open source licence, and has received over 1500 download requests to date.

### Reasoning

A key aspect of DAML + OIL is its well-defined formal semantics (**http://www.daml.org/2001/03/model-theoretic-semantics.html**) [15]. This provides an account of exactly how we should interpret composite expressions or descriptions in the language, and facilitates machine interpretation of DAML + OIL ontologies. For example, DAML + OIL contains different restriction types representing existential and universal quantification, allowing us to be explicit about representing, for example, the class of proteins which can bind to *some* DNA but can also bind to other things, or alternatively, the

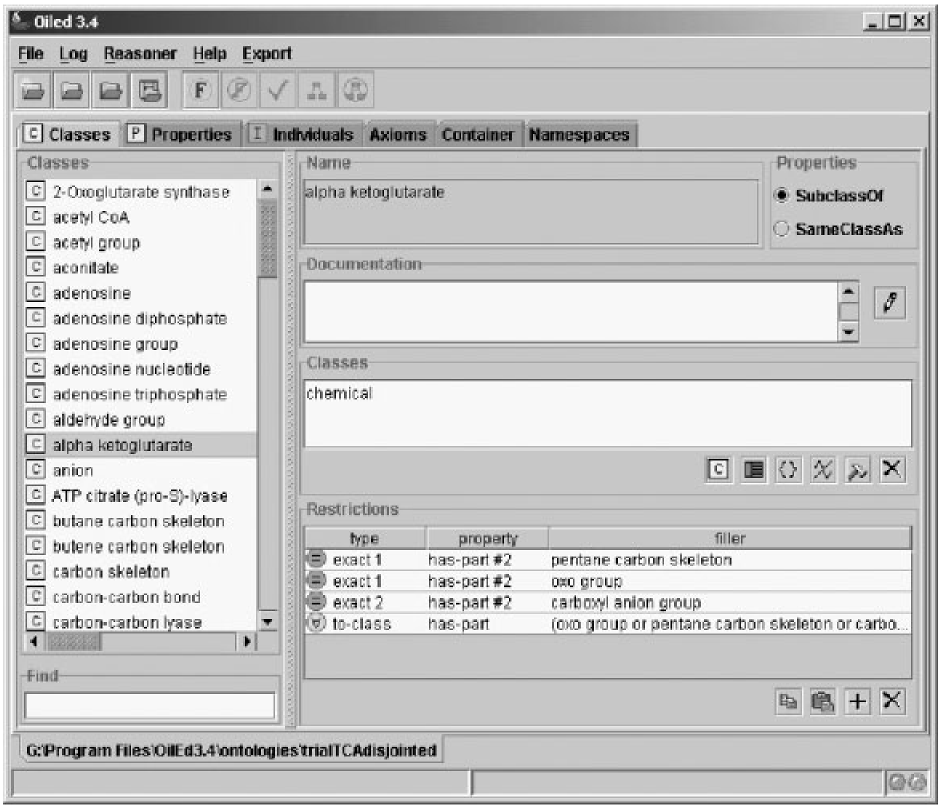

**Figure 2.** OilEd class of proteins which *only* bind to DNA and not to other things.

These semantics then allow us to employ *reasoners* to infer relationships between classes. In particular, we can infer *subsumption*, or 'kind-of' links between class descriptions, and thus build classification hierarchies which are based precisely on the semantics of the descriptions applied to classes. In addition, *satisfiability* testing allows us to determine when class definitions are unsatisfiable or incoherent, i.e. when no instances of the class could possibly exist.

OilEd Uses a reasoner [the current version uses the FaCT (**http://www.cs.man.ac.uk/fact**) [16] DL reasoner] to organize concept hierarchies. The ontology is translated to an equivalent DL model, and the reasoner is then used to build a classification hierarchy of the concepts in the model.

This use of a reasoner is a useful addition to the ontologist's toolbox, particularly during the construction and maintenance of ontologies. The task of ontology integration can also be supported with reasoning — cross-ontology relationships can be defined, with the reasoner assisting in spotting *equivalences* between concept definitions in different ontologies. This is an approach that has proved successful in the context of integrating database schemas (e.g. ICOM [17]).

### Normalization and modularization of ontologies

So, we have an expressive knowledge representation language, an editor for building an ontology and the possibility of using machine support for reasoning with such an ontology. We have already described how handcrafting ontologies can become difficult as they become large and complex. We offer a way of avoiding such problems, but at the cost of adding further complexity. We need guidelines on how to use such representations and support. A logical mess is no better than a hand-crafted mess. Figure 1 shows the kind of complexity that may arise in a DAML + OIL ontology. We need to avoid unmanageable complexity and make sure our descriptions are not 'tangled'; we do this by *normalizing* the parts of our ontology into simple modules that can then be combined to give the complex descriptions in which we are interested.

Just because we describe our concepts using logic expressions does not in itself deliver 'good' ontologies. It is a truism that logic guarantees only that truth follows from truth. Logic says nothing about what follows from falsehood, neither can a logic engine make deductions from any information unless it is explicitly represented. This means that to use logic you must tell 'the truth, the whole truth, and nothing but the truth'. DL-based ontologies always cover wider ground than the application to which they will be put. In order to describe enzymes, we also need to describe reactions, substrates, products and co-factors. The 'whole truth' is always bigger than any model. All models, even logical models, are approximations.

There are two commonly occurring problems: (a) not telling the truth; and (b) not telling the whole truth. Not telling the truth — lying to the system — happens most frequently when the system is not expressive enough to say what we want to say. This is less of a problem with DAML + OIL, as it has a great expressivity when compared with previous representations. We are, for example, able to say that a GPCR must have only seven transmembrane regions, but having seven such regions does not necessarily make something a GPCR. Despite this expressivity, 'lying' still occurs. The other reason for not telling the truth is blunder and confusion. A more expressive language makes this, if anything, more likely because there are many ways to say any one thing, and the consequences of 'clever' solutions can be hard to determine. So the first purpose of normalization is to 'keep it simple'.

The second problem, not telling the whole truth, is much more difficult to manage. It is extremely easy to leave out information or, even more pernicious, to represent it only in the names, comments, and perhaps conventions. It is obvious to any reader why 'protease' falls under both 'protein' and 'enzyme', that one hierarchical link has to do with structure, the other with function. But how is this to be represented to a logic engine? What if we want to take out one structure ontology and plug in another, new, improved version? What if we want to extend the detail of protein structure and keep the same structure of biological function, or vice versa? Normalization provides a means of distinguishing the reasons for classification and imposes a discipline that makes it much more likely that authors will make all relevant information explicit.

Copyright © 2003 John Wiley & Sons, Ltd. 

At the same time, normalization makes it possible to take ontologies apart into pieces and put them back together again. To do this logically, the 'joins' have to be explicit and the sections must not overlap.

What normalization does is to turn all multiple classification over to the logic engine. Then, if the reason for classification is not explicit, the classification does not happen — which is usually easy to spot and fix. A minimal skeleton of simple trees is still required — you have to start some where — but for everything else sufficient information must be represented so that the classifier can make the correct inferences.

So we transform the process of developing a complex richly interconnected multiple hierarchy into the much simpler task of creating many smaller, simpler hierarchies. Each simple hierarchy has to be a simple tree, i.e. each concept in the skeleton has only one parent in the skeleton. Then we provide the descriptions and definitions that links the simple hierarchies and let the logic engine infer the complex structure. If something is missing in the definitions or descriptions, it is usually the case that some concept appears grossly out of place, usually much too far up the classification, where it stands out. If something is inconsistent, then the reasons for the inconsistency will be explicit and the logic engine will mark it. The result is a simple, consistent structure which we can explain to others, maintain and extend.

### Migrating ontologies to a property-based form: Gong

We can see an instance of this normalization in the GONG project, where DAML + OIL is being used to give reasoning-based support to the development of GO. To make descriptions of GO terms we must, for example, add ontologies of chemicals in order to make descriptions of enzyme and metabolism terms. The growing size and complexity of GO is forcing its curators to spend more and more time on the mundane task of maintaining the consistency and completeness of its internal structure. The GONG project aims to demonstrate that, in principle, migrating to a finer-grained formal conceptualization in DAML + OIL will allow computational techniques such as DLs to aid in the curation and delivery of the ontology and so allow the curators to focus on curating the biological knowledge. Providing a detailed conceptualization of every GO concept is a large task, and so GONG aims to take a staged approach. Small inroads will be taken in order to solve specific problems. As more formal definitions are produced, the reasoner can be used more frequently to give support to the manual curation task.

The first specific task for GONG is helping to maintain the metabolism section of the existing GO. Within GO many concepts have multiple parents. That is because many concepts can be sensibly grouped in more than one manner. The maintenance of 'this-is-a' links is a manual process. Experience from the medical domain has shown that numerous parent–child links are omitted in such hand-crafted, phrase-based controlled vocabularies [7]. While of less importance to manual interpretation, machine interpretation will falter in the face of such inconsistencies.

Take, for example, the metabolism concepts. The majority of metabolism concepts within the GO describe a metabolic process acting on a chemical molecule, e.g. heparin biosynthesis. This concept can be grouped by the nature of the process; heparin biosynthesis is a kind of heparin metabolism because biosynthesis is a particular kind of metabolism. The concept can also be grouped by the nature of the chemical being processed; heparin biosynthesis is a kind of peptidoglycan biosynthesis, because heparin belongs to the class of peptidoglycans. Figure 3 shows how the concept heparin biosynthesis was organized in the July 2002 version of GO.

However, additional parent relationships are possible and their inclusion does affect the retrieval of information using the GO. In fact, heparin is more specifically a kind of glycosaminoglycan, and so heparin biosynthesis could sensibly be placed as a kind of glycosaminoglycan biosynthesis. Addition of 'this-is-a' link now allows extra gene products to be returned from a query asking for all gene products that have the biological process concept 'glycosaminoglycan biosynthesis', because by extension they will include gene products annotated with the process concept 'heparin biosynthesis'.

How can we use DL to maintain the links automatically? First, we dissect the concept, explicitly

Copyright © 2003 John Wiley & Sons, Ltd. 

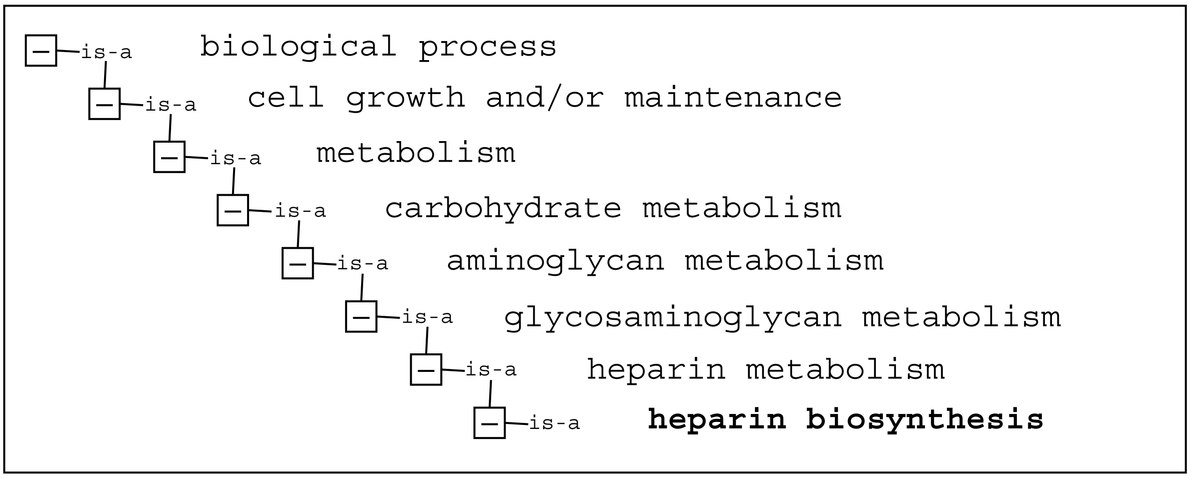

**Figure 3.** Directed acyclic graph showing position of heparin biosynthesis in the original Gene Ontology (July 2002)

stating the concept's definition in a formal representation. This provides the substrate for DL reasoners to infer new 'is-a' links and remove redundant links. Within a large phrased-based ontology, such as GO, which contains many concepts within a narrow semantic range, it is possible to use automated techniques to construct candidate dissections by simply parsing the term names.

For example, many metabolism terms in the GO follow the pattern, 'chemical name followed by either metabolism or catabolism or biosynthesis'. If a term name fits this pattern, a dissection can be created from the relevant phrase constituents. Table 1 shows the DAML + OIL definitions for heparin biosynthesis and glycosaminoglycan biosynthesis.

The process of dissection breaks down the existing concept into more elemental concepts related together in a formal semantic manner. These elemental concepts rarely exist in the original ontology and so themselves have to be defined. These are the hand-crafted, single axial taxonomies described in the normalization stage in the section on Normalization and Modularization of ontogenies, above. The nature of these elemental definitions can range from a simple taxonomy to complex dissections in their own right. Knowledge is fractal, and the decision about 'how far down to model' is based on the degree of knowledge the final ontology needs to support. For example, GO is not used to annotate information concerning detailed chemical atomic substructure, and so modelling that

**Table 1.** Human readable DAML + OIL definitions for heparin biosynthesis and glycosaminoglycan biosynthesis

| | |
|---|---|
| Concept name | heparin biosynthesis |
| DAML + OIL definition | **class** heparin biosynthesis **defined**<br>**subClassOf** biosynthesis<br>**restriction onProperty** acts_on **hasClass** heparin<br>(**acts_on** is a **unique** property) |
| Paraphrase | biosynthesis which acts solely on heparin |
| Concept name | glycosaminoglycan biosynthesis |
| DAML + OIL definition | **class** glycosaminoglycan biosynthesis **defined**<br>**subClassOf** biosynthesis<br>**restriction onProperty** acts_on **hasClass** glycosaminoglycan |
| Paraphrase | biosynthesis which acts solely on glycosaminoglycans |

Copyright © 2003 John Wiley & Sons, Ltd. 

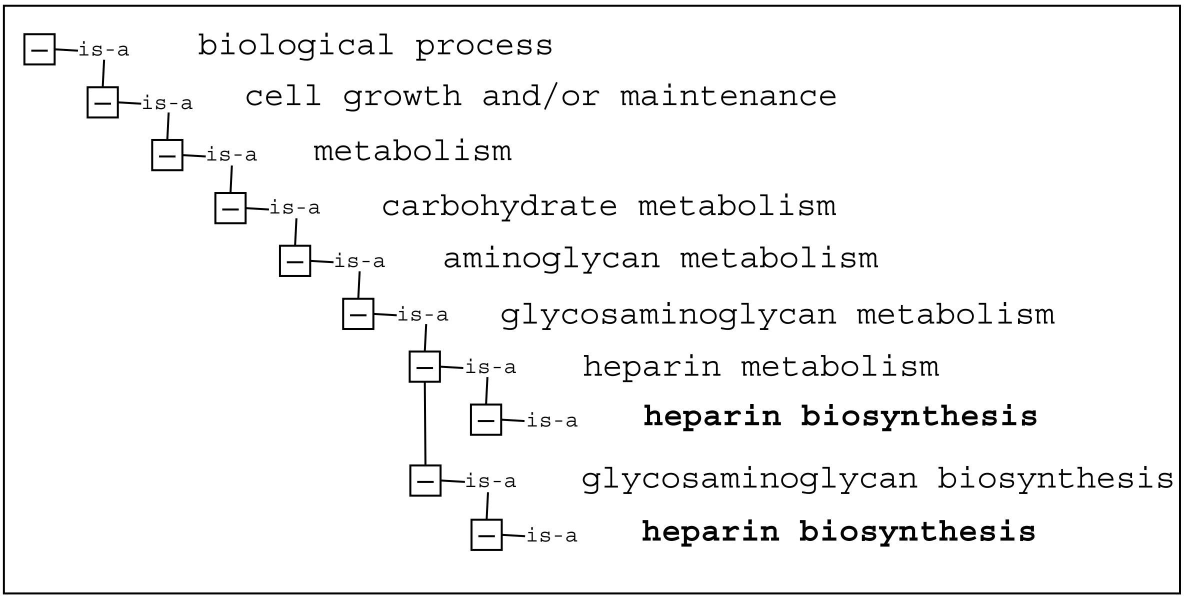

**Figure 4.** Directed acyclic graph showing additional parent for heparin biosynthesis found using the reasoner knowledge would be fruitless at this stage. Once all the explicit terminological knowledge is in place, we can submit it to the reasoner and examine the report of the new subsumption links it has inferred, what links are redundant, and what concept definitions are logically inconsistent.

Reassuringly, the changes reported by the DL reasoner represent mostly additional relationships hard to spot by human eye, and not errors in biological knowledge.

For example, the reasoner reported that heparin biosynthesis has a new 'is-a' parent, 'glycosaminoglycan biosynthesis'. Figure 4 shows the hierarchy that results from these descriptions being submitted to the reasoner; it can be compared with Figure 3. These reports can then be sent to the editorial team for comment and action if necessary. Although the nature of the changes made by the reasoner are limited and may not be accepted by the GO team as the correct solution, in terms of the biology, they are helpful in pointing out areas of the ontology which may need attention. For example, inferring a new subsumption relationship to a very general concept may point out the need for new ontology fragments incorporating new intermediate concepts. The reasoner reported that 'taurine catabolism' GO0019529 has new super 'catabolism' GO0009056. This led to the GO editor to actually make 'taurine catabolism' a child of the more specific term, 'amino acid derivative catabolism; GO0042219' instead. The editor then created a new term, 'taurine biosynthesis; GO0042412' and made it a child of both 'taurine metabolism; GO0019530' and 'amino acid derivative biosynthesis; GO0042398'.

Although the GONG project has only tackled a small portion of GO, it has shown the utility of such an approach. In our first experiment, we migrated 350 metabolism terms to the DAML + OIL, property-based form. From these descriptions, 22 missing 'is-a' relationships were found. This means that nearly one in ten concepts in the hand-crafted form of GO were not completely described. This does not mean that GO is bad or wrong — it is another demonstration that building ontologies is difficult. As already mentioned, few real biological errors have been found — most were errors of omission. We feel that we have already begun to show the utility of an approach in which it is possible to use one knowledge representation language to both represent a hand-crafted ontology and migrate it *in situ* to a more expressive, property-based form.

### Discussion

In this article we have presented an approach to representing ontologies used in the Bioinformatics

Copyright © 2003 John Wiley & Sons, Ltd. 

and Medical Informatics groups at the University of Mancherster. We are now using DAML + OIL as our knowledge representation language. This allows us to use ontologies represented in a variety of forms, from hand-crafted taxonomies of phrases to full-blown concept definitions based upon the use of a concept's properties. In the latter form, DL-based reasoners can be used to infer the classification encoded within the descriptions of a concept's properties.

Such a DAML + OIL-based representation allows ontologies to be as simple as possible, yet as complex as necessary. In addition, we can migrate from the simpler form to the more complex, reasoning-supported form without having to throw away the simpler representation. Due to this range of entry levels, it is possible to include experts in the domain, rather than in DL's, in the process of constructing DAML + OIL ontologies, allowing exploitation of their specialist knowledge.

Although with tools such as OilEd and the reasoners it encompasses, we have the foundations of a full ontology development environment and methodology, much work remains to be done. As well as the extensions needed for OilEd described above, some barriers still remain to non-specialist use of DAML + OIL. Using the full expressivity of DAML + OIL is still difficult, particularly the use of elements such as universal and existential quantification. GONG has begun to develop methods and tools to support the migration of terms to property descriptions through automated dissections. However, techniques that will allow high-expressivity not to be a barrier between expert and representation still need to be developed and are an active area of research. Nevertheless, we feel that the approach advocated at Manchester forms the basis for moving arguments from 'What is an ontology?' to 'How do we best deliver ontologies that serve the purposes we require?'

### Acknowledgements

Chris Wroe is supported by the myGrid e-Science pilot (EPSRC GR/R67743) and GONG (DARPA DAML subcontract PY-1149 from Stanford University). Phil Lord is also supported by the myGrid project. Sean Bechhofer is supported by EU Grant IST-2001-33052. We would like to thank Midori Harris and Jane Lomax of the GO editorial team for their advice and for responding to the reports.

### References

1. Rogers J, Roberts A, Solomon D, *et al*. 2001. GALEN ten years on: tasks and supporting tools. In *MedInfo 2001 Studies in Health Technology and Informatics*, Haux R, Rogers R, Patel V (eds). IOS Press: Amsterdam; 256–260.
2. Goble CA, Stevens R, Ng G, *et al*. 2001. Transparent Access to Multiple Bioinformatics Information Sources. *IBM Systems, (special issue on deep computing for the life sciences)* **40**(2): 532–552.
3. Wroe C, Stevens RD, Goble CA, Roberts A, Greenwood M. 2003. A suite of DAML + OIL Ontologies to describe bioinformatics services and data. *Int J Coop Inf Syst* (accepted for publication).
4. Wroe CJ, Stevens RD, Goble CA, Ashburner M. A methodology to migrate the Gene Ontology to a description logic environment using DAML + OIL. 8th Pacific Symposium on Biocomputing (PSB) (accepted for presentation, 2003).
5. The Gene Ontology Consortium. 2000. Gene Ontology: tool for the unification of biology. *Nature Genet* **25**: 25–29.
6. World Health Organization. 1992. *International Statistical Classification of Diseases and Related Health Problems*, 10th revision (ICD-10). World Health Organization: Geneva.
7. Rogers JE, Price C, Rector AL, Solomon WD, Smejko N. 1998. Validating clinical terminology structures: integration and cross-validation of Read Thesaurus and GALEN. In *AMIA Fall Symposium*, Lake Buena Vista, FL, USA.
8. Swiss-PROT Keywords: **http://www.expasy.ch/cgi-bin/keywlist.pL**
9. Baader F, McGuinness D, Nardi D, Schneider PP (eds). 2003. *The Description Logic Handbook: Theory, Implementation and Applications*. Cambridge University Press: Cambridge.
10. Horrocks I. 2002. DAML + OIL: a reason-able web ontology language. In *Proc. EDBT 2002*. Lecture Notes in Computer Science. Springer-Verlag, Heidelberg, 2–13.
11. Stevens R, Horrocks I, Goble C, Bechhofer S. 2001. Building a reasonable bioinformatics ontology using OIL. In *Proceedings of the IJCAI 2001 Workshop on Ontologies and Information Sharing*, 81–90.
12. OilEd: **http://oiled.man.ac.uk**
13. GONG: **http://gong.man.ac.uk**
14. myGrid: **http://mygrid.man.ac.uk**
15. DAML + OIL semantics: **http://www.damL.org/2001/03/model-theoretic-semantics.htmL**
16. FaCT: **http://www.cs.man.ac.uk/fact**
17. Franconi E, Ng G. 2000. The i.com tool for intelligent conceptual modelling. In *7th Intl. Workshop on Knowledge Representation meets Databases (KRDB'00).*

Copyright © 2003 John Wiley & Sons, Ltd.
